# Memristor–CMOS Hybrid Circuits Implementing Event-Driven Neural Networks for Dynamic Vision Sensor Camera

**DOI:** 10.3390/mi15040426

**Published:** 2024-03-22

**Authors:** Rina Yoon, Seokjin Oh, Seungmyeong Cho, Kyeong-Sik Min

**Affiliations:** School of Electrical Engineering, Kookmin University, Seoul 02707, Republic of Korea; flsk0419@kookmin.ac.kr (R.Y.);

**Keywords:** memristors, memristor–CMOS hybrid circuits, event-driven neural networks, dynamic vision sensor cameras

## Abstract

For processing streaming events from a Dynamic Vision Sensor camera, two types of neural networks can be considered. One are spiking neural networks, where simple spike-based computation is suitable for low-power consumption, but the discontinuity in spikes can make the training complicated in terms of hardware. The other one are digital Complementary Metal Oxide Semiconductor (CMOS)-based neural networks that can be trained directly using the normal backpropagation algorithm. However, the hardware and energy overhead can be significantly large, because all streaming events must be accumulated and converted into histogram data, which requires a large amount of memory such as SRAM. In this paper, to combine the spike-based operation with the normal backpropagation algorithm, memristor–CMOS hybrid circuits are proposed for implementing event-driven neural networks in hardware. The proposed hybrid circuits are composed of input neurons, synaptic crossbars, hidden/output neurons, and a neural network’s controller. Firstly, the input neurons perform preprocessing for the DVS camera’s events. The events are converted to histogram data using very simple memristor-based latches in the input neurons. After preprocessing the events, the converted histogram data are delivered to an ANN implemented using synaptic memristor crossbars. The memristor crossbars can perform low-power Multiply–Accumulate (MAC) calculations according to the memristor’s current–voltage relationship. The hidden and output neurons can convert the crossbar’s column currents to the output voltages according to the Rectified Linear Unit (ReLU) activation function. The neural network’s controller adjusts the MAC calculation frequency according to the workload of the event computation. Moreover, the controller can disable the MAC calculation clock automatically to minimize unnecessary power consumption. The proposed hybrid circuits have been verified by circuit simulation for several event-based datasets such as POKER-DVS and MNIST-DVS. The circuit simulation results indicate that the neural network’s performance proposed in this paper is degraded by as low as 0.5% while saving as much as 79% in power consumption for POKER-DVS. The recognition rate of the proposed scheme is lower by 0.75% compared to the conventional one, for the MNIST-DVS dataset. In spite of this little loss, the power consumption can be reduced by as much as 75% for the proposed scheme.

## 1. Introduction

Spiking neural networks (SNNs) have been studied for decades for mimicking the energy-efficient cognition and intelligence of the human brain [1,2,3,4]. One of the key features of SNNs is a spike-based operation, which is believed a key factor able to realize the brain’s low-power consumption [5,6,7,8,9]. Since neurons can only react to spike signals that are temporarily active, spike-based communication among neurons in the human brain may be helpful for developing energy-efficient computing. One more advantage of spike-based operation is that the timing information of spike signals can be used. In the SNNs, the information is delivered by spikes, which are represented with streaming events with time. If some events are redundant, they can be skipped, relieving them from involvement in the neural network’s operation. By doing so, more power consumption can be saved compared to the conventional neural networks that rely on the static data [10,11].

Though the spike signals are beneficial for saving power consumption, the discontinuity found in spike signals makes it difficult to train the SNNs directly using the conventional backpropagation method [1,4,6,12]. To circumvent the training problem from the discontinuity of spikes, some methods such as surrogate gradient learning have been investigated [13,14]. Though the surrogate gradient method is successful in achieving the excellent performance of neural networks, the benefits of SNNs such as their low-power consumption and simple hardware architecture can be diminished because backpropagation through time (BPTT) with the surrogate gradient model is very complicated in terms of hardware implementation and it may waste too much energy for the training [9,15,16].

The alternative method for processing streaming events such as spikes is to implement artificial neural networks (ANNs) integrated into CMOS digital circuits [17,18,19,20]. One major advantage of ANNs for streaming events is that the normal backpropagation method can be directly used unlike spiking neural networks. The conventional digital CMOS neural networks have been shown to perform in processing streaming events [21]. However, the hardware and power overheads were large [22]. This is because the streaming data should be accumulated over time and converted to histogram data in conventional neural networks. To do so, a large-size memory circuit such as large SRAM should be used to store all the accumulated events [23]. Moreover, the frequent data movement between the memory and logic circuits consumes a large amount of energy every time the ANNs perform computation [19,24].

To take the benefits from both SNNs and ANNs explained earlier, memristor–CMOS hybrid circuits are proposed in this paper, for implementing event-driven neural networks, which can be trained directly by conventional backpropagation. Memristors are nonvolatile memories that can retain the stored data even after power-off [25]. The memristor’s current–voltage relationship can be used in performing the energy-efficient matrix multiplication compared to digital CMOS multiplier circuits [26,27]. Moreover, memristors can be fabricated on top of CMOS devices [28]. By doing so, the memristor–CMOS hybrid circuits can be integrated on a single substrate to minimize the interconnection overhead between the memristors and CMOS devices.

**Figure 1:**
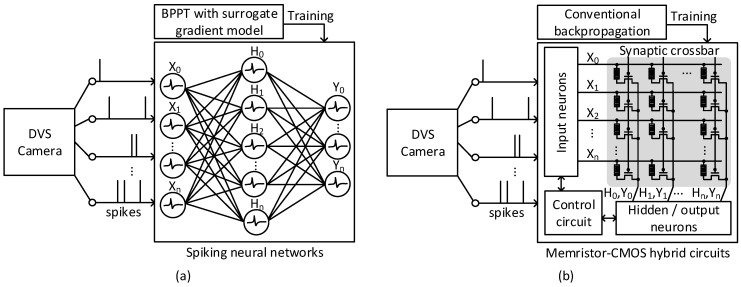
(**a**) The spiking neural networks trained by backpropagation through time with surrogate gradient model for processing the spike events from DVS camera [29]; (**b**) the memristor–CMOS hybrid circuits of event-driven neural networks trained by conventional backpropagation for processing the spike-events from DVS camera.

Before explaining the memristor–CMOS hybrid circuits of event-driven neural networks, first, a traditional SNN architecture is shown in Figure 1a [29]. Here, a DVS camera generates streaming events with time. The generated events enter the SNN architecture as indicated in Figure 1a. The SNN should be trained by the complicated backpropagation modified for the SNN with the surrogate gradient model [13,14]. To carry this out, the SNN architecture should be implemented in complicated hardware and its power consumption can be large. The event-driven ANN architecture that was trained with the conventional backpropagation method is shown as the alternative approach in Figure 1b. Here, the ANN’s hardware can be implemented with the memristor–CMOS hybrid circuits. By doing so, the complicated digital CMOS circuits can be avoided in order to make the event-driven ANN circuit in Figure 1b energy-efficient.

Explaining Figure 1b specifically, the proposed event-driven ANN is composed of an input neuron block, a synaptic memristor–crossbar block, a hidden/output neuron block, and a controller. At the input neurons, spiking events are converted to histogram data, using simple memristor-based latches, as explained in detail in the next section. The synaptic memristor crossbar can perform energy-efficient multiplication–accumulation (MAC) calculation, using the current–voltage relationship of memristors [27,30]. The hidden and output neurons convert the crossbar’s column currents to the output voltages according to the activation function [31]. The control circuit proposed in this paper is important to make the event-driven neural networks energy-efficient. The controller can adjust the MAC calculation frequency according to the workload of streaming events. If the streaming events are generated very frequently, the controller increases the MAC calculation frequency to a high value to increase the computation capability. On the other hand, the events are generated rarely with time, and the MAC calculation frequency is adjusted to a low value to minimize the MAC computing power. Moreover, the controller can terminate the MAC calculation by itself by turning on the self-exit signal. If the streaming events generated from the DVS camera are redundant, the unnecessary MAC calculation is terminated automatically.

In Section 2, the memristor–CMOS hybrid circuits of event-driven neural networks are explained in detail. In Section 3, simulation results are indicated and discussed for MNIST-DVS and POKER-DVS datasets. The neural network’s performance and power consumption are tested and verified for the proposed event-driven ANN circuits. In Section 4, we finally summarize this paper.

## 2. Method

The DVS camera’s operation is based on the neural system’s motion detection principle, which is similar to that of the biological brain [32,33]. The DVS camera as a brain-mimicking one can detect and process rapid movements effectively while maintaining energy efficiency. The DVS camera has a wide dynamic range and minimal power consumption, making it easy to efficiently process and understand streaming video data.

**Figure 2:**
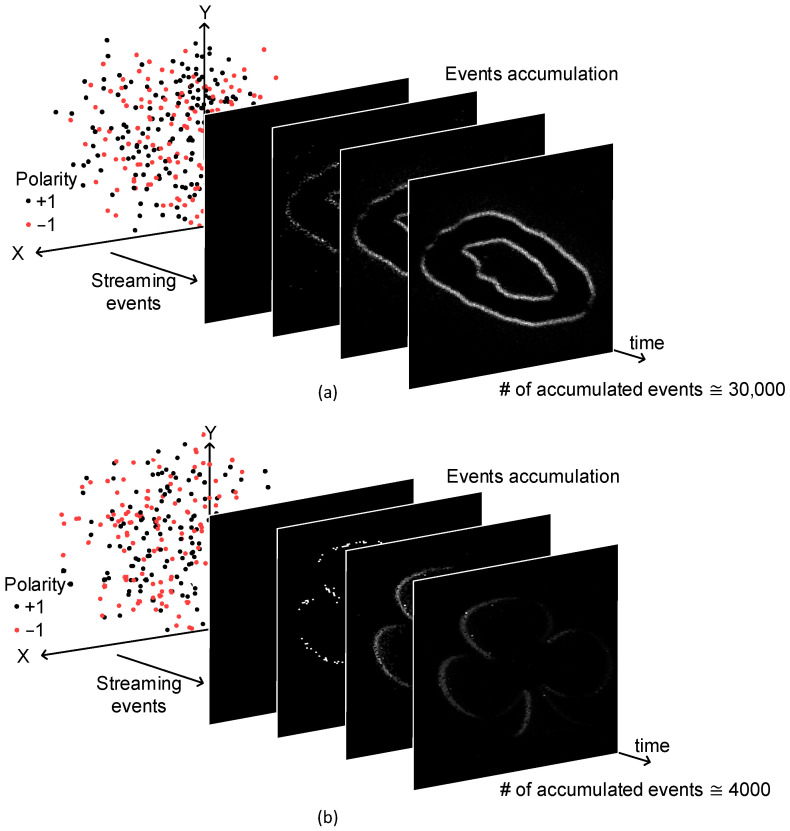
(**a**) The streaming events of MNIST-DVS dataset accumulated with time; (**b**) the streaming events of POKER-DVS dataset accumulated with time.

Streaming events from a DVS camera are shown in Figure 2a for the MNIST-DVS dataset. Here, the DVS camera’s events are plotted discretely in time. Each event’s information is composed of that event’s time, x-coordinate, y-coordinate, and polarity. The x-coordinate and y-coordinate are the event’s location. The polarity represents whether the light intensity is increased or not. The captured events can accumulate with time, as shown in Figure 2a. Here, the total number of events of digit ‘0’ accumulated with time becomes as many as about 30,000 when the capturing time is as long as 2.9 s. The pixel resolution of the DVS camera is 128 × 128. One thing to note is that the number of events within a given time period depends on the movement speed. If the object moves fast, the number of events generated from the DVS camera should be larger than when the object moves slowly. Figure 2b shows streaming events of the POKER-DVS dataset accumulated with time. Here, the number of accumulated events reaches around 4000.

**Figure 3:**
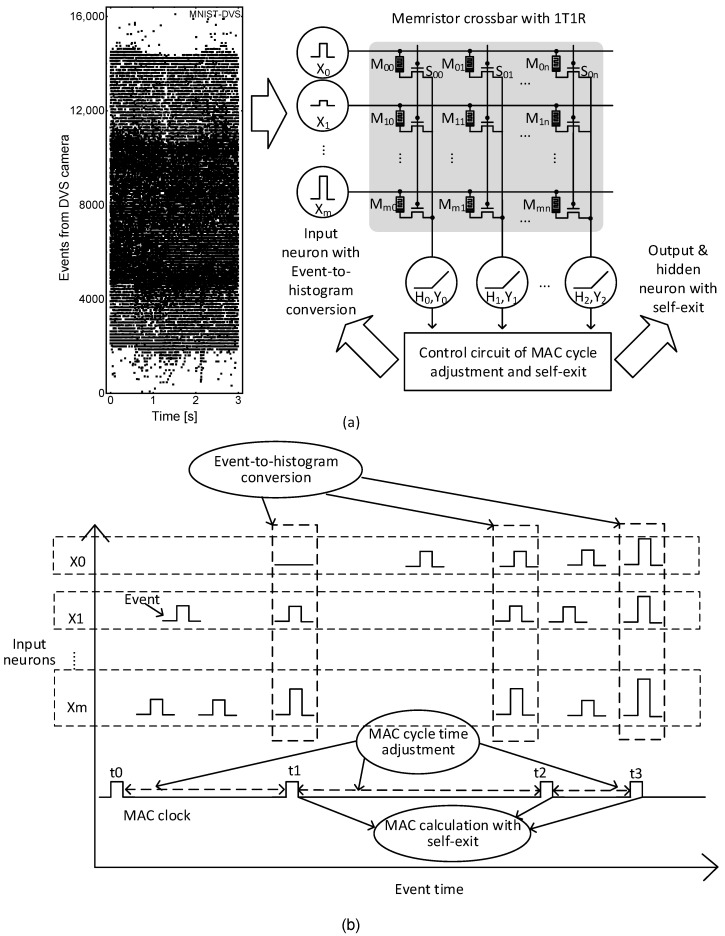
(**a**) The block diagram of memristor–CMOS hybrid circuits for processing DVS camera’s events; (**b**) the timing diagram of memristor–CMOS hybrid circuits for explaining MAC clock adjustment and self-exit.

Figure 3a shows a block diagram of memristor–CMOS hybrid circuits for processing streaming events from the DVS camera. In Figure 3a, streaming events are shown on the left. The memristor–CMOS hybrid circuits of event-driven neural networks proposed in this paper are shown on the right of Figure 3a. In the hybrid circuits, a synaptic memristor crossbar is found in the middle. The crossbar’s rows and columns are connected with input and hidden/output neurons, respectively. On the bottom, there is a controller circuit that adjusts the MAC calculation frequency and performs self-exit to reduce power consumption.

The operation is explained as follows. The event dataset is generated by the DVS camera in Figure 3a as time increases, and the streaming events generated discretely in time are plotted as black dots. The events generated discretely in time enter the input neurons, where the events captured by the DVS camera are converted to histogram data, as shown in Figure 3a. The input neurons are represented with X0, X1, and Xm. Here, ‘m’ is the number of input neurons. The converted histogram data in the input neurons are represented by voltages. The voltages are applied to the synaptic memristor crossbar, as indicated in Figure 3a. In the crossbar, memristor cells represent synaptic weights and each memristor’s current can be calculated with Ohm’s law. The crossbar’s column current is the current summation from memristor cells belonging to the column. The current summation is equal to the column’s MAC calculation result, which can be fed to the hidden/output neurons. The hidden/output neurons represented with Y0, Y1, and Yn can calculate the activation function. Here, ‘n’ is the number of hidden/output neurons. In Figure 3a, the control circuit can monitor the collective information of activation functions from the hidden and output neurons. If the collective information is enough to make a decision with the input streaming events, the controller can turn on the self-exit signal to disable the MAC calculation clock. By doing so, the MAC calculation can be terminated immediately to avoid the unnecessary processing of redundant input events. One more thing to note is that the control circuit can also adjust the MAC calculation frequency according to the workload of streaming events. When many events are generated frequently from the DVS camera, the MAC calculation clock becomes fast. On the other hand, if the events are rare, the MAC clock becomes slow, with the workload of event processing being decreased.

Figure 3b shows a timing diagram of memristor–CMOS hybrid circuits for processing event data from the DVS camera. Here, input neurons such as X1, X2, and Xm are shown on the *y*-axis. Events generated from the DVS camera enter the input neurons. The events shown on the first row in Figure 3b enter the input neuron, X0, with time increasing. The events on the second row are for the input neuron, X1. On the bottom, an MAC clock shown in Figure 3b is activated at t0, t1, t2, and t3 by the controller. When the MAC clock is activated at t1, the MAC calculation is performed by the hybrid circuits for the histogram of events accumulated during t0–t1. At t2, the MAC calculation is performed for the histogram data during t0–t2. Similarly, at t3, the histogram data from t0 to t3 are used in the MAC calculation. One more thing to note is that the MAC clock can be disabled by turning on the self-exit flag and adjusted according to the workload of input events, in order to save unnecessary power consumption, as mentioned earlier.

**Figure 4:**
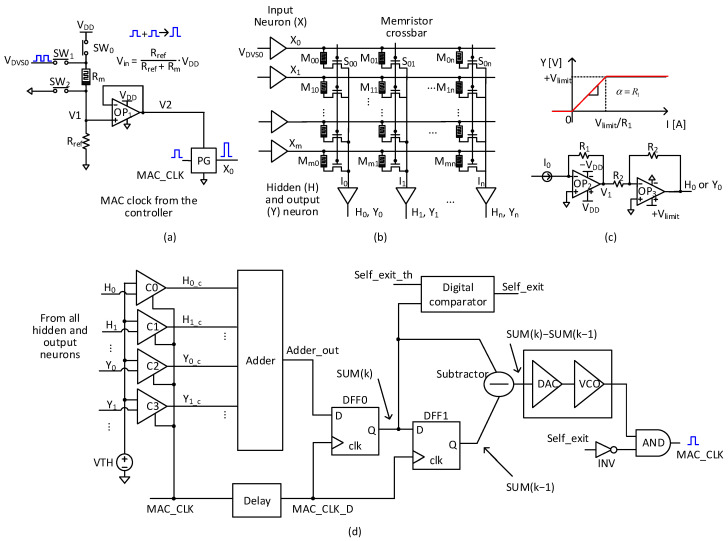
(**a**) The input neuron circuit with event-to-histogram conversion; (**b**) the synaptic memristor crossbar; (**c**) the hidden/output neuron circuit with ReLU activation function; (**d**) the control circuit of MAC frequency adjustment and self-exit.

Figure 4a shows an input neuron circuit with event-to-histogram conversion. In Figure 4a, Rm is a memristor and Rref is a reference resistor. SW0, SW1, and SW2 represent controlling switches. VDVS0 represents spiking events with time. V1 is a resistor divider’s voltage between Rm and Rref. V1 enters a voltage buffer of OP1. V2 is its output voltage. V2 drives a pulse generator of PG in Figure 4a. PG’s input is an MAC clock signal of ‘MAC_CLK’. PG’s output is X0, which enters the synaptic crossbar in Figure 4b.

The operation of Figure 4a is as follows. When ‘MAC_CLK’ is low, SW0 is open and SW1 and SW2 are closed. At this time, if the events enter the input neuron, Rm’s conductance is increased gradually according to the number of input events. When ‘MAC_CLK’ is high, SW0 is closed and SW1 and SW2 are open. The gradual change in Rm’s conductance takes place when ‘MAC_CLK’ is low and can increase V1 according to the resistor divider’s rule of Rref/(Rref + Rm), for the MAC calculation time when ‘MAC_CLK’ is high. The increased V1 can modulate PG’s output, X0, according to the number of events received by the input neuron until then. By doing so, X0’s amplitude can represent histogram information calculated from the number of spiking events from the DVS camera until that time.

Figure 4b shows a synaptic memristor crossbar. The crossbar’s rows are applied from X0 and X1 to Xm, which are the modulated voltages from the input neurons in Figure 4a. Here, M00 and S00 work as 1T-1M cells. I0, I1, and In are the crossbar’s column currents. In Figure 4b, Y0, Y1, and Yn are output voltages generated from output neurons. Similarly, H0, H1, and Hn represent output voltages from hidden neurons. The memristor crossbar’s columns can be connected to both hidden and output neurons, which are indicated in Figure 4b.

Figure 4c shows a hidden/output neuron circuit with an ReLU activation function. I0 means the crossbar’s column current. Here, the crossbar’s column current, I0, is converted to a hidden/output neuron’s voltage, H0 or Y0, through OP2 and OP3, as shown in Figure 4c. The OP2 and OP3 compose a current-to-voltage converter with R1 and R2. One thing to note here is that the converted voltage should be limited by Vlimit, as shown in Figure 4c. The activation function indicated in Figure 4c is similar to the ReLU function used in most artificial neural networks.

Figure 4d shows a control circuit for MAC frequency adjustment and self-exit. Here H0, H1, Y0, and Y1 are voltages from the hidden and output neurons of Figure 4c. C0, C1, C2, and C3 are analog comparators, where H0, H1, Y0, and Y1 are compared with an activation threshold voltage of VTH. One should note that the comparators are activated only when ‘MAC_CLK’ is high. They are deactivated to not consume any power when ‘MAC_CLK’ is low. H0_C, H1_C, Y0_C, and Y1_C are the comparator’s digital outputs that are 0 or 1. If H0 is larger than VTH, H0_C becomes 1. Otherwise, H0_C becomes 0. The comparator’s digital outputs enter the adder, as indicated in Figure 4d. The adder’s output, ‘Adder_out’ in Figure 4d, means the number of hidden and output neurons that have their activation voltages exceeding VTH.

In Figure 4d, ‘Adder_out’ enters DFF0 sampled by ‘MAC_CLK_D’. Here ‘MAC_CLK_D’ means a delayed signal of ‘MAC_CLK’. ‘MAC_CLK_D’ is used in Figure 4d to capture ‘Adder_out’ after the MAC calculation when ‘MAC_CLK’ is high. DFF0’s output is connected to DFF1. By doing so, DFF0 and DFF1 can store ‘SUM(k)’ and ‘SUM(k − 1)’ simultaneously. Here, ‘SUM(k)’ and ‘SUM(k − 1)’ represent present and previous values of ‘Adder_out’, as shown in Figure 4d. The difference between ‘SUM(k)’ and ‘SUM(k − 1)’ enters a Digital-to-Analog Converter (DAC). If the difference is large, the DAC’s output voltage becomes large, too. The DAC’s output voltage is applied to a Voltage-Controlled Oscillator (VCO). If the DAC’s output voltage is large, the ‘MAC_CLK’ frequency becomes high. On the other hand, if the DAC’s output voltage is small, the VCO lowers the ‘MAC_CLK’ frequency.

In Figure 4d, ‘Self_exit’ means a flag signal that is turned on when ‘SUM(k)’ is larger than ‘Self_exit_th’. ‘Self_exit_th’ means a threshold number for measuring the activation density of hidden/output neurons. If the number of hidden/output neurons activated exceeds a number defined by ‘Self_exit_th’, the ‘Self_exit’ flag signal is turned on from the digital comparator, in Figure 4d. The ‘Self-exit’ signal enters an inverter of ‘INV’ and then the inverter’s output enters an AND gate of ‘AND’. If the ‘Self_exit’ signal is high, ‘MAC_CLK’ is disabled. Once the ‘MAC_CLK’ is disabled, the memristor–CMOS hybrid circuits in Figure 4 can avoid further unnecessary power consumption.

**Figure 5:**
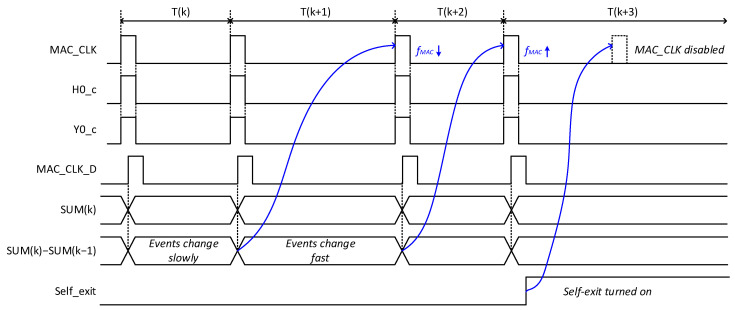
The waveforms of signals in the control circuit of MAC frequency adjustment and self-exit.

Figure 5 shows waveforms of signals in the control circuit of MAC frequency adjustment and self-exit explained in Figure 4d. Here ‘MAC_CLK’ represents an MAC calculation clock signal, which activates the analog comparator’s operation of C0, C1, C2, and C3 in Figure 4d. ‘H0_C’ and ‘Y0_C’ are the comparator’s outputs. When ‘MAC_CLK’ is high, the ‘H0_C’ and ‘Y0_C’ are generated from the comparators. They enter the adder, and the calculated summations are stored at DFF0 and DFF1 in Figure 4d at a rising edge of ‘MAC_CLK_D’, as shown in Figure 5. Here, ‘MAC_CLK_D’ is the delayed signal of ‘MAC_CLK’. As explained earlier, SUM(k) and SUM(k − 1) in Figure 5 represent the present and previous values of ‘Adder_out’. The difference between ‘SUM(k)’ and ‘SUM(k − 1)’ becomes large if the DVS camera’s events change fast. Otherwise, if the DVS camera’s events change slowly, the difference becomes small. As a result, the frequency of ‘MAC_CLK’ can be modulated according to the difference between ‘SUM(k)’ and ‘SUM(k − 1)’, as shown in Figure 5. For self-exit generation, if the number of activated hidden and output neurons exceeds a number defined by ‘Self-exit_th’, the ‘Self-exit’ signal becomes high. Once ‘Self-exit’ becomes high, ‘MAC_CLK’ is disabled as indicated in Figure 5.

**Figure 6:**
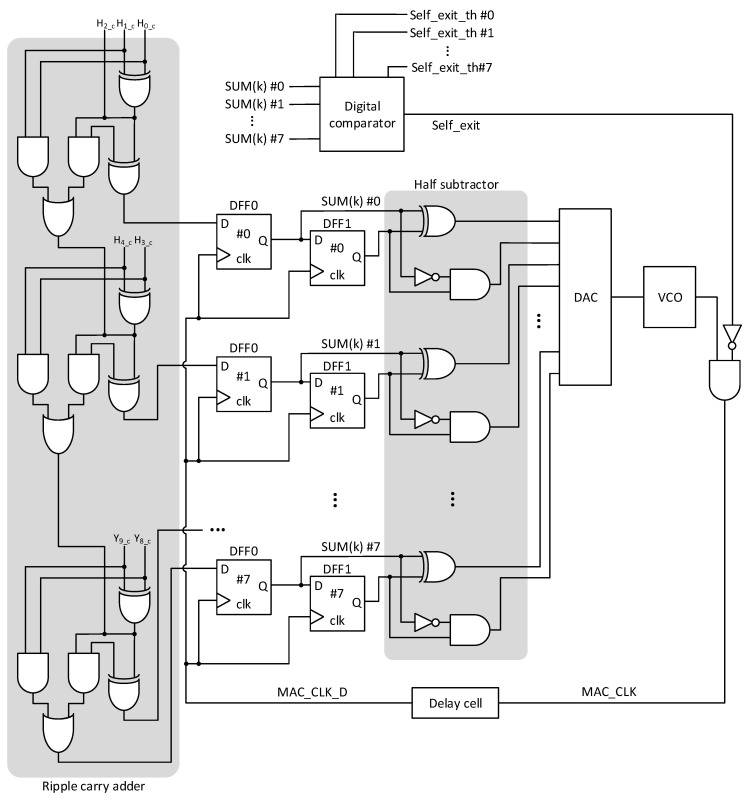
The circuit schematic of the neural network’s controller for MAC frequency adjustment and self-exit functions.

A digital part of the neural network’s controller in Figure 4d is shown in Figure 6. Basically, the circuits shown in Figure 6 are designed by a Generic PDK of 0.18 um CMOS technology provided from CADENCE CAD software (Virtuoso version 6.1.8-64b, and Spectre version 20.1.0.068) [34]. The circuits shown in Figure 6 are scalable basically because they are designed by common CMOS design techniques. With little effort, the circuits in Figure 6 can be re-designed by smaller CMOS technology such as 0.13 um. For simulating the DAC and VCO in Figure 6, they are modeled by the Verilog-A language for describing the analog circuit’s behaviors in the circuit simulation [35]. The behavioral models of DAC and VCO are obtained from the real measurement results from the fabricated chips [36,37].

## 3. Results

Figure 6 shows butterfly curves of memristors from the measurement and Verilog-A model [38,39]. The inset in Figure 6 shows a cross-sectional view of the memristors measured, where Mn_2_O_3_ is used as a memristive film. Ta and Pt form top and bottom electrodes, respectively, as shown in Figure 6. For the measurement in Figure 6, a Keithley 4200 (Solon, OH, USA) (a semiconductor parameter analyzer) is used in this paper. The Verilog-A model used in Figure 6 shows good agreement with the measurement [40]. The total error between the measurement and the simulation model is calculated within 3.3% for the entire operational region of memristors [38]. Details of the Verilog-A model and the fabrication of memristors measured in Figure 6 can be found in the references [38,39].

**Figure 7:**
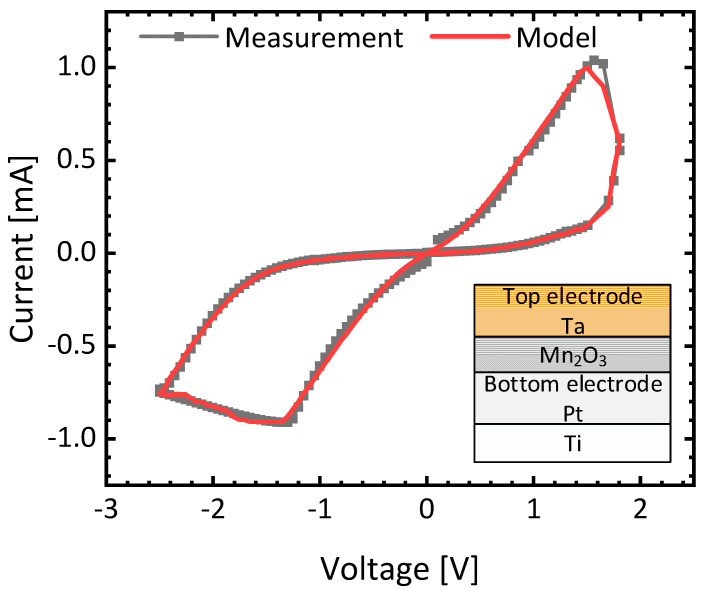
The butterfly curves of memristors with measurement and Verilog-A model [38,39]. The inset figure shows a cross-sectional view of memristors measured in this figure.

For verifying the hybrid circuits of memristor crossbars and CMOS neurons and controllers, the circuit-level simulation was used in this paper. The circuit-level simulation is performed using CADENCE SPECTRE, where the memristor’s Verilog-A model and 0.18 CMOS circuits are combined for the circuit simulation of the entire neural network. As mentioned, the memristor’s Verilog-A model is obtained from the measurement [38]. The 0.18 um CMOS spike models are obtained from the Generic 018 CMOS PDK of CADENCE [34]. One more thing to note is that the DAC and VCO used in Figure 7 are also modeled using Verilog-A for saving the simulation time and the models are from the chip measurement [36,37].

**Figure 8:**
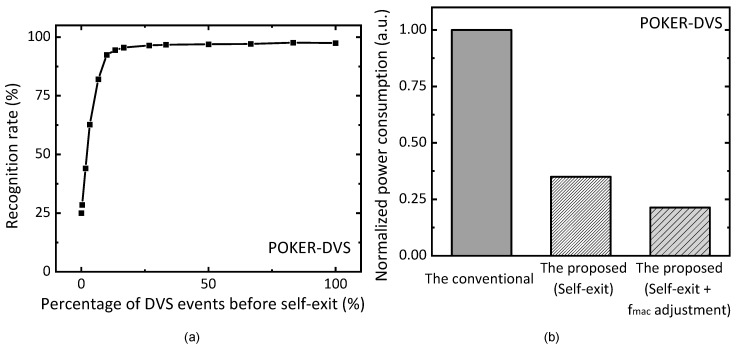
(**a**) The simulated recognition rate of POKER-DVS dataset with increasing percentage of DVS events before the self-exit; (**b**) the normalized power consumption of the conventional scheme, the proposed scheme with self-exit, and the proposed scheme with self-exit and MAC frequency adjustment.

Figure 8a shows a simulation result of the recognition rate tested for the POKER-DVS dataset. The dataset is composed of four symbols of card decks. The total numbers of training and test images in the POKER-DVS dataset are 4480 and 1099, respectively. In Figure 8a, the *x*-axis represents a percentage of DVS events before the self-exit flag is turned on. At the starting time, the number of events generated from the DVS camera is 0. At this time, the recognition rate is as low as 25%. This is because POKER-DVS is composed of only four symbols. As the percentage of DVS events trained by the proposed memristor–CMOS hybrid circuits of event-driven neural networks becomes larger, the recognition rate improves. If the percentage of DVS events reaches 30%, the recognition rate seems almost saturated around the recognition rate of 96.95%. When the percentage of DVS events is 100%, the recognition rate can be as high as 97.45%. A rate as high as 100% means that all the events are received from the DVS camera. Figure 8b compares a normalized power consumption of neural networks for three schemes. They are the conventional scheme, the proposed scheme with only self-exit, and the proposed scheme with both self-exit and MAC frequency adjustment, respectively. The proposed scheme with only self-exit can save 61% in power consumption compared to the conventional scheme without self-exit and MAC frequency adjustment. If the proposed scheme uses both self-exit and MAC frequency adjustment, it can reduce the power consumption by 79% compared to the conventional one.

**Figure 9:**
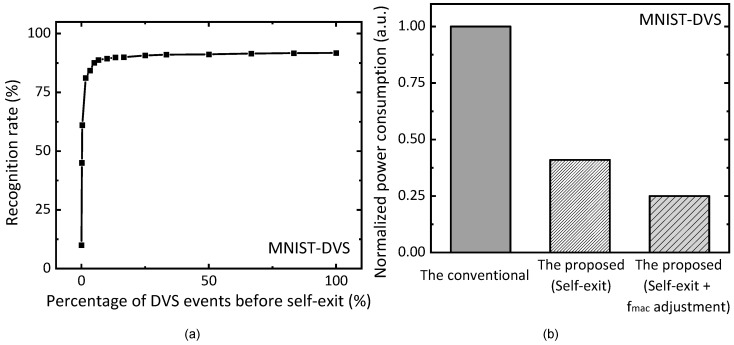
(**a**) The simulated recognition rate of MNIST-DVS dataset with increasing percentage of DVS events before the self-exit; (**b**) the normalized power consumption of the conventional scheme, the proposed scheme with only self-exit, and the proposed scheme with both self-exit and MAC frequency adjustment.

Figure 9a shows a simulation result of recognition rate tested for the MNIST-DVS dataset. The dataset of MNIST-DVS is composed of ten digits of 0–9. The total numbers of training and test images are 8000 and 2000, respectively. In Figure 9a, the *x*-axis represents a percentage of DVS events before self-exit. At the starting time of DVS events, the number of events received from the DVS camera is 0. At this time, the recognition rate is as low as 10%. This is because the MNIST-DVS dataset is composed of 10 digits of 0–9. As the percentage of DVS events becomes larger, the recognition rate becomes better. If the percentage of DVS events reaches 30%, the recognition rate seems almost saturated about 91.05%. When the percentage of DVS events is 100%, the recognition rate is as high as 91.8%.

The recognition rate of the MNIST-DVS dataset can be improved better, if the number of layers and hidden neurons are increased more. For 91.8%, the number of layers is only 2 and the number of hidden neurons is 256. If the number of layers becomes 3 and the numbers of the first and second hidden neurons are 500 and 500, respectively, the recognition rate is increased from 91.8% to 93.7%. This 93.7% is very comparable to 95.2%, which is the highest number reported for the MNIST-DVS dataset [20].

Figure 9b shows a normalized power consumption of neural networks for the conventional scheme, the proposed scheme with only self-exit, and the proposed scheme with both self-exit and MAC frequency adjustment. The proposed scheme with only self-exit can save 59% in power consumption compared to the conventional scheme that does not use self-exit and MAC frequency adjustment. If the proposed scheme uses both self-exit and MAC frequency adjustment, it can reduce the power consumption more by 75% compared to the conventional one.

Finally, the gesture DVS dataset should be mentioned [19]. The gesture DVS dataset is composed of 11 classes of different gestures and the data of each gesture have streaming events according to gestures moving with increasing time. One particular thing to note is that the previous POKER-DVS and MNIST-DVS dataset are events recorded for static images not moving objects. The proposed hybrid circuits of memristor crossbars and CMOS neurons and controllers are also simulated for the gesture DVS dataset. The simulation result indicates that the proposed hybrid circuits can recognize the gestures as high as 70.5%. If the number of layers becomes 3 and the numbers of the first and second hidden neurons are 500 and 500, respectively, the recognition rate increases from 70.5% to 73.66%. This 73.66% is very comparable to the 74.1% reported previously [17]. The reported recognition rate of 74.1% was obtained from the CNN-based neural network trained by the normal backpropagation algorithm [17]. Here, CNN represents Convolutional Neural Networks. If Backpropagation Through Time (BPTT) with the surrogate model can train spiking neural networks, the recognition rate of the gesture DVS dataset can be as high as 93.64% [41]. This improvement in spiking neural networks is mainly because BPTT can optimize the neural networks better for temporal information than normal backpropagation. But, as mentioned earlier in the introduction, BPTT and the surrogate model need very complicated hardware to implement the spiking neural networks. The proposed hybrid circuits of synaptic memristor crossbars and CMOS neurons and controllers can make hardware implementation much simpler than the spiking neural networks with BPTT and the surrogate.

## 4. Conclusions

For processing streaming events from a Dynamic Vision Sensor camera, two types of neural networks can be considered. One are spiking neural networks, where simple spike-based computation is suitable for low-power consumption, but the discontinuity in spikes can make the training complicated in terms of hardware. The other one are digital CMOS-based neural networks that can be trained directly using the normal backpropagation algorithm. However, the hardware and energy overhead can be significantly large, because all streaming events must be accumulated and converted into histogram data, which requires a large amount of memory such as SRAM.

In this paper, to combine the spike-based operation with the normal backpropagation, memristor–CMOS hybrid circuits are proposed for implementing event-driven neural networks in hardware. The proposed hybrid circuits are composed of input neurons, synaptic crossbars, hidden/output neurons, and a neural network’s controller. In the input neurons, the DVS camera’s events can be preprocessed. The events are converted to histogram data using very simple memristor-based latches. After preprocessing the events, the histogram data are delivered to the ANN, which can be implemented by synaptic memristor crossbars. The memristor crossbars can perform low-power MAC calculation according to the current–voltage relationship of memristors. The hidden/output neurons can convert the crossbar’s column currents to the output voltages according to the ReLU activation function. The neural network’s controller adjusts the MAC calculation frequency according to the workload of event computation. Moreover, the controller can disable the MAC calculation clock automatically to minimize the unnecessary power consumption.

The proposed hybrid circuits have been verified by circuit simulation for several event-based datasets such as POKER-DVS and MNIST-DVS. The circuit simulation results indicate that the neural network’s performance proposed in this paper is degraded by as low as 0.5% while saving as much as 79% in power consumption for POKER-DVS. The recognition rate of the proposed scheme is lower by 0.75% compared to the conventional one, for the MNIST-DVS dataset. In spite of this little loss, the power consumption can be reduced by as much as 75% for the proposed scheme.

## Data Availability

Dataset available on request from the authors.
